# A Rare Cause of Massive Upper Gastrointestinal Hemorrhage in Immunocompromised Host

**DOI:** 10.4021/gr400w

**Published:** 2012-01-20

**Authors:** Obai Abdullah, Nicole A. Pele, Yumei Fu, Imran Ashraf, Murtaza Arif, Matthew L. Bechtold, Ajitinder Grewal, Hazem T. Hammad

**Affiliations:** a Department of Internal Medicine, University of Missouri - Columbia, MO, USA; b Department of Pathology, University of Missouri - Columbia, MO, USA; c Division of Gastroenterology and Hepatology, University of Missouri - Columbia, MO, USA

**Keywords:** Invasive mucurmycosis, Gastrointestinal bleed

## Abstract

Mucormycosis is an invasive and aggressive opportunistic fungal infection that usually presents with rhinocerebral or pulmonary involvement and rarely involves the gastrointestinal tract. The disease is acute with mortality rate up to 100%. A 68-year-old male was undergoing treatment at a local hospital for COPD exacerbation with IV steroids and antibiotics. Two weeks into his treatment he suddenly developed massive upper GI bleeding and hemodynamic instability that necessitated transfer to our tertiary care hospital for further treatment and management. An urgent upper endoscopy revealed multiple large and deep gastric and duodenal bulb ulcers with stigmata of recent bleeding. The ulcers were treated endoscopically. Biopsies showed fibrinopurulent debris with fungal organisms. Stains highlighted slightly irregular hyphae with rare septa and yeast suspicious for Candida. The patient was subsequently placed on fluconazole. Unfortunately, the patient’s general condition continued to worsen and he developed multiorgan failure and died. Autopsy revealed disseminated systemic mucormycosis. Most of the cases of gastrointestinal mucormycosis were reported from the tropics and few were reported in the United States. The disease occurs most frequently in immunocompromised individuals. The rare incidence of GI involvement, acute nature, severity and the problematic identification of the organisms on biopsies make antemortem diagnosis challenging. Treatment includes parenteral antifungals and debridement of the infected tissues. Gastroenterologists should be aware of this rare cause of gastrointestinal bleeding and understand the importance of communication with the reviewing pathologist so that appropriate, and often lifesaving, therapies can be administered in a timely manner.

## Introduction

Mucormycosis is an invasive and aggressive opportunistic fungal infection that usually presents with rhinocerebral or pulmonary involvement and rarely involves the gastrointestinal tract. The disease is acute with mortality rate up to 100%. Herein we describe a rare case of disseminated invasive mucormycosis that presented with massive upper GI bleeding.

## Case Report

A 68-year-old male was undergoing treatment at a local hospital for COPD exacerbation with IV steroids and antibiotics. Two weeks into his treatment he suddenly developed massive upper GI bleeding and hemodynamic instability that necessitated transfer to our tertiary care hospital for further treatment and management.

An urgent upper endoscopy revealed multiple large and deep gastric and duodenal bulb ulcers with stigmata of recent bleeding. The ulcers were treated endoscopically with epinephrine injection and bipolar cautery, and proton pump inhibitor therapy was initiated. Biopsies obtained from the gastric mucosa showed fibrinopurulent debris with fungal organisms. Periodic acid-Schiff and Grocott’s methanamine silver stains were performed which highlighted slightly irregular hyphae with rare septa and yeast suspicious for *Candida*. The patient was subsequently placed on fluconazole.

**Figure 1:**
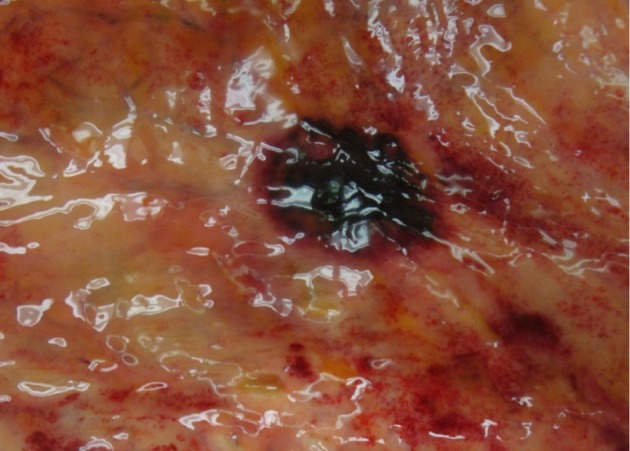
Autopsy on this 68 year-old patient showing hemorrhagic ulceration in the stomach.

**Figure 2:**
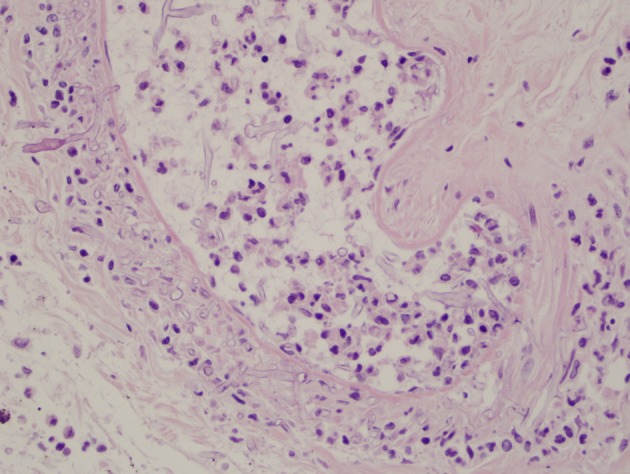
Angioinvasive fungal organisms in the vascular channel and the vessel walls of the postmortem stomach, 400 × H&E.

Unfortunately, the patient’s general condition continued to worsen and he developed multiorgan failure including: altered mental status, respiratory failure, thyroid storm, atrial fibrillation, renal failure, and pancytopenia. He died 2 weeks later due to multiorgan failure at which time an autopsy revealed disseminated systemic mucormycosis with necrotizing hemorrhagic infarctions in the stomach (Fig.1, 2), lungs, kidneys, thyroid, and brain.

## Discussion

Mucormycosis refers to several different diseases caused by fungi in the order Mucorales, which includes the species Mucor, Rhizopus, Absidia and Cunninghamella [1, 2], ubiquitous saprophytic molds found in soil and organic matter worldwide [3]. These rare fungal infections usually only occur in individuals with impaired immunity, diabetes mellitus, hematological or solid-organ malignancies, transplantation, neutropenia and steroid therapy [4].

The most common clinical presentation of mucormycosis is rhinocerebral [5] and pulmonary involvement [6]. Gastrointestinal infection with invasive mucor­mycosis is extremely rare [7]. All portions of the gastrointestinal tract can be involved, with the stomach, ileum, and colon most commonly affected. The organisms probably enter the host by way of the mouth [8]. Disseminated disease might occur and affect almost all body organs as in our case [9].

When gastrointestinal tract is involved, mucormycosis can present with nonspecific symptoms such as abdominal pain, nausea, and vomiting [10] ; however, given its angioinvasive abilities, invasion of blood vessels can occur leading to hemorrhage and perforation as in our case [7, 11]. This clinical picture, along with epigastric distention, is the most frequent presentation [12]. Mortality of patients with GI mucormycosis is very high [12]. In a meta-analysis study of 929 cases of mucormycosis, mortality rate was 85% in patients with GI involvement [13].

Endoscopically, gastric mucormycosis can have the appearance of a white, fibrinous growth [7] or be associated with hemorrhage and necrosis [10]. A typical gastrointestinal lesion consists of a dark ulcer with sharply demarcated edges and with necrosis and thrombosis in adjacent vessels [14, 15]. Examination of the biopsy specimen with direct light without staining can sometimes establish the diagnosis within minutes after the procedure [16]. The organism has a characteristic morphology consisting of irregular, broad, ribbon-like nonseptate hyphae which branch at right-angles and can be seen invading tissue and vascular channels [17]. On occasion, the ribbon-like hyphae can twist and fold, resembling septa; and rarely true septa are present [18]. Angioinvasion was not identified in our endoscopic biopsies which may have been due to the limited size and superficial nature of the specimen.

Diagnosis is challenging, especially on scant tissue samples, and is complicated by distortion of the organism during tissue retrieval and processing. Significant pathological expertise is required to make the diagnosis [19]. If there is a strong suspicion of Mucorales infection, this information should be conveyed to the pathologist at the time of evaluation. The diagnosis is usually made by biopsy of the suspected area during surgery, endoscopy, or commonly postmortem at autopsy [7].

Hofman et al. [20] observed the usefulness of frozen section in rhinocerebral mucormycosis diagnosis and management on seven patients. In his observation, cytological imprints performed on surgical biopsies were thought to be less effective than frozen section for the immediate diagnosis of mucormycosis because of the presence of hemorrhages and necrotic tissue. However, in three of seven cases, typical Mucorales organisms stained by toluidine blue, haematoxylin and eosin, or Gomori Grocott’s methanamine silver method could be cytologically demonstrated.

Interpretation of tissue cultures in these patients is a complicated and slow process. The organism is a common laboratory contaminant and may be destroyed during tissue processing rendering false positive and negative results, respectively [21]. Organisms in tissue specimens with histopathologically identified mucormycosis often fail to grow in fungal cultures [19]. Roden et al. [13] reviewed 929 cases of reported mucormycosis between 1940 and 2003. In his review, only 50% culture positive patients were identified.

Most of the cases of gastrointestinal mucormycosis were reported from the tropics; however, the disease is increas­ingly becoming an iatrogenic and/or nosocomial problem in industrialized nations [7] and recent studies have reported dramatic increase in the incidence of mucormycosis [22-24]. Very few cases of gastrointestinal mucormycosis affecting adults have been reported in the United States [6, 7, 25].

Systemic amphotericin B is the mainstay of treatment. Reversal of underlying medical disease and often surgical debridement of the infected tissues are necessary for successful management [12].

### Conclusion

Most of the cases of gastrointestinal mucormycosis were reported from the tropics and few were reported in the United States. The disease occurs most frequently in immunocompromised individuals. The rare incidence of GI involvement, acute nature, severity and the problematic identification of the organisms on biopsies make antemortem diagnosis challenging. Treatment includes parenteral antifungals (amphotericin B) and debridement of the infected tissues. Advances in medical therapies have improved survival for many disease states which have resulted in an ever increasing number of immunocompromised patients. It is likely that we will see a corresponding increase in the incidence of Mucormycosis. Gastroenterologists should be aware of this rare cause of bleeding gastric and duodenal ulcerations and understand the importance of communication with the reviewing pathologist so that appropriate, and often lifesaving, therapies can be administered in a timely manner. 
